# Applicability of droplet digital polymerase chain reaction for minimal residual disease monitoring in Philadelphia‐positive acute lymphoblastic leukaemia

**DOI:** 10.1002/hon.2913

**Published:** 2021-08-16

**Authors:** Michela Ansuinelli, Irene Della Starza, Alessia Lauretti, Loredana Elia, Veronica Siravo, Monica Messina, Lucia Anna De Novi, Akram Taherinasab, Martina Canichella, Anna Guarini, Robin Foà, Sabina Chiaretti

**Affiliations:** ^1^ Hematology, Department of Translational and Precision Medicine Sapienza University Rome Italy; ^2^ GIMEMA Foundation Rome Italy; ^3^ Department of Molecular Medicine Sapienza University Rome Italy

**Keywords:** ddPCR, MRD, Ph+ acute lymphoblastic leukemia

## Abstract

In Ph+ acute lymphoblastic leukaemia (Ph+ ALL), minimal residual disease (MRD) is the most relevant prognostic factor. Currently, its evaluation is based on quantitative real‐time polymerase chain reaction (Q‐RT‐PCR). Digital droplet PCR (ddPCR) was successfully applied to several haematological malignancies. We analyzed 98 samples from 40 Ph+ ALL cases, the majority enrolled in the GIMEMA LAL2116 trial: 10 diagnostic samples and 88 follow‐up samples, mostly focusing on positive non‐quantifiable (PNQ) or negative samples by Q‐RT‐PCR to investigate the value of ddPCR for MRD monitoring. DdPCR *BCR/ABL1* assay showed good sensitivity and accuracy to detect low levels of transcripts, with a high rate of reproducibility. The analysis of PNQ or negative cases by Q‐RT‐PCR revealed that ddPCR increased the proportion of quantifiable samples (*p* < 0.0001). Indeed, 29/54 PNQ samples (53.7%) proved positive and quantifiable by ddPCR, whereas 13 (24.1%) were confirmed as PNQ by ddPCR and 12 (22.2%) proved negative. Among 24 Q‐RT‐PCR‐negative samples, 13 (54.1%) were confirmed negative, four (16.7%) resulted PNQ and seven (29.2%) proved positive and quantifiable by ddPCR. Four of 5 patients, evaluated at different time points, who were negative by Q‐RT‐PCR and positive by ddPCR experienced a relapse. DdPCR appears useful for MRD monitoring in adult Ph+ ALL.

## 1 INTRODUCTION

The t (9; 22) (q34; q11.2)—resulting in the formation of the Philadelphia (Ph) chromosome—which encodes for the *BCR*/*ABL1* fusion protein, causes a constitutive tyrosine kinase activation and interaction with other transforming elements.^1^ In acute lymphoblastic leukaemia (ALL), its incidence increases with age: it is detected in 25‐30% of adults and in about 50% of elderly patients.^2^ Currently, management of Ph+ ALL is based on: (i) the identification of the *BCR‐ABL1* rearrangement by karyotyping, FISH and/or quantitative real‐time polymerase chain reaction (Q‐RT‐PCR); (ii) an induction therapy based on tyrosine kinase inhibitors (TKI) with^3^, ^4^, ^5^, ^6^, ^7^ or without chemotherapy^8^, ^9^, ^10^, ^11^, ^12^, ^13^; (iii) an accurate minimal residual disease (MRD) monitoring to evaluate the response to therapy, to personalize treatment and predict relapse.^14^, ^15^, ^16^, ^17^, ^18^ Since the outcome of adult patients with Ph+ ALL has greatly improved following the introduction of TKIs and may be further improved by combining this class of compounds with immunotherapeutic strategies,^12^ MRD negativity must be considered the primary goal of treatment.

Indeed, MRD is regarded as the strongest independent prognostic factor both in Ph+ and Ph− ALL. In the latter, MRD can be detected by multiparametric flow cytometry and molecular methods, such as PCR amplification‐based methods that use leukaemia‐specific (fusion gene transcripts) or patient‐specific (immunoglobulin/T‐cell receptor (IG/TR) gene rearrangements) molecular markers,^19^, ^20^, ^21^ which represent the gold‐standard technique for MRD assessment. Recently, in addition to these ‘conventional methods’, new techniques, namely digital droplet PCR (ddPCR) and next generation sequencing (NGS),^22^, ^23^, ^24^, ^25^, ^26^ have been explored, showing an overall higher sensibility in anticipating a relapse.

DdPCR, a third generation PCR, might represent a valid alternative to Q‐RT‐PCR also for BCR/ABL1 quantification: it is based on a water‐oil emulsion which determines the parcellization of the sample into at least 20,000 droplets and then PCR amplification is carried out within each droplet. This technique is highly sensitive and accurate, and it does not require a reference curve; furthermore, it has affordable costs and an easy interpretation of results. ddPCR improves the limit of detection (LOD) and quantification^27^, ^28^, ^29^, ^30^ of Q‐RT‐PCR. While this assay has proven valuable compared to Q‐RT‐PCR in lymphoma and Ph− ALL, its role has been scarcely investigated in Ph+ ALL. In this study, we compared Q‐RT‐PCR and ddPCR in Ph+ ALL, focusing on the set up of the BCR/ABL1 assay and on the evaluation of the specificity and sensitivity of the method.

## 2 MATERIALS AND METHODS

### 2.1 Determination of cDNA input

For cDNA synthesis, 1 μg of RNA was reverse‐transcribed by using the SuperScript VILO cDNA Synthesis Kit (Invitrogen™), the same approach used to perform Q‐RT‐PCR. To define the optimal cDNA input, we tested three different amounts: 1, 2.5 and 5 μL. In order to simulate a MRD condition, we diluted the cDNA of the diagnostic material in cDNA obtained from mononuclear cells (MNCs) from healthy donors to produce five serial dilutions (10^−1^, 10^−2^, 10^−3^, 10^−4^, 10^−5^). MNC were obtained after‐Ficoll density gradient separation. Each experiment was carried out including negative controls: for every condition tested, a no template control (NTC) and MNC from healthy donors was analyzed at least in triplicate.

### 2.2 Evaluation of limit of detection, specificity and reproducibility

In order to define the (limit of detection, LOD) for the *BCR*/*ABL1* p190 and p210 assays, we tested serial dilutions of the lowest point (10 copies) of a plasmid standard curve built on five plasmid 10‐fold dilutions (10^6^, 10^5^, 10^3^, 10^2^ and 10 copies) (Werfen‐Instrumentation, Bedford Laboratories, Bedford, OH): thus, we obtained 5, 2, 1 and 0.5 copies, corresponding to 1 × 10^−4^; 5 × 10^−5^; 1 × 10^−5^; 5 × 10^−6^; 1 × 10^−6^, in a logarithmic scale. These dilutions were escalated into an increasing number of replicates for each dilution point: two replicates for 1 × 10^−4^; six replicates for 5 × 10^−5^; eight replicates for 1 × 10^−5^; 12 replicates for 5 × 10^−6^ and 14 replicates for 1 × 10^−6^. The replicates copy number of each dilution point were then merged to generate a final definitive value, allowing to obtain a greater depth of analysis. To evaluate the specificity of the assay, four pools of MNC obtained from healthy donors (donor A + B; donor C + D; donor E + F; donor G + H) and NTC controls were examined in a dedicated plate. In particular, 32 NTC replicates and 16 replicates of each pool were tested. To assess the reproducibility of the assay, we analyzed three diagnostic Ph+ ALL samples and their 10^−1^, 10^−2^ and 10^−3^ dilutions. Each condition was run in duplicate and we compared the results obtained for each replicate. The same samples were repeated in two independent experiments.

## 3 DdPCR CONDITIONS

To perform our experiments, we used the same primers and probes of Q‐RT‐PCR, according to the BIOMED1 recommendations^31^, ^32^ and we followed the Biorad protocols. Probes for target BCR/ABL1 p190 and p210 were labelled with FAM and BHQ1 reporters and probe for control gene was labelled with FAM and HEX reporters. Primers and probes were used at a final concentration of 900 nmol/L and 250 nmol/L, respectively. In each experiment we included negative controls, that is, MNCs, NTCs and a Ph− ALL diagnostic sample; as positive control samples, we used the first and the last dilution point of the plasmid curve, randomly distributed in the plate. The ABL1 gene was used as control gene to evaluate the quality of material and to calculate the ratio between the target and control gene. The reaction mixture, containing the cDNA, ddPCR Supermix (Bio‐Rad), primers and probes were loaded into the DG8 cartridge wells, covered with the DG8 gasket, and loaded into the QX200 Droplets Generator together with 70 μL of droplet oil, in order to generate the droplets. During droplet generation, template molecules are distributed randomly into droplets. Due to the random nature of the partitioning, the fluorescence data after amplification are well fit by a Poisson distribution, thus, it can be used to determine the number of template molecules in a droplet given the fluorescence data. After droplets generation, they were transferred from the DG8 cartridge into a 96‐well PCR plate which was sealed with a Bio‐Rad pierceable foil heat seal and subsequently amplified through a Bio‐Rad Thermal Cycler GeneAmp PCR System 9700 (Applied Biosystems). Thermal‐cycling conditions were the following: one cycle at 95°C for 10 min, 40 cycles at 94°C for 30 s, 40 cycles at 60°C for 1 min, one cycle at 98°C for 10 min and 4°C as holding temperature. Finally, the PCR plate was loaded into the QX200 Droplets Reader and data analyzed by using the QuantaSoft analysis Software version 1.7.4. According to the manufacturer's instructions, only analyses giving a number of droplets ≥9000/replicate were considered acceptable; the correct quantification of each experiment was carried out by setting by manual curation a threshold value with a sufficient distance from the background to ensure suitable sensitivity and specificity, as described by the manufacturer's application.

For ddPCR results, interpretation we followed the guidelines proposed within the EuroMRD Consortium^26^ (supporting information).

Results were expressed as [copies/μl of the target gene]/[copies/μl of the control gene] × 100. The concentration—indicated as the number of copies/μl—is provided by the QuantaSoft software.

### 3.1 Population of study

After having established the cDNA input and after having established the sensitivity and the specificity of the assay, we focused on the screening of cases enrolled mainly in the GIMEMA LAL2116 (*n* = 36), by analyzing cDNA from 10 diagnostic samples and 88 follow‐up samples derived from bone marrow (BM) of adult Ph+ ALL patients, including 10 samples classified as positive, 54 classified as positive non‐quantifiable (PNQ) and 24 classified as negative by Q‐RT‐PCR analysis. In the GIMEMA LAL2116 protocol, the evaluation of MRD was performed according by Q‐RT‐PCR.^12^ Four additional cases, not enrolled in the above mentioned protocol, were also evaluated (1 PNQ and 3 negative samples). More information about study population can be found in supporting information.

## 4 RESULTS

### 4.1 Determination of cDNA input

**FIGURE 1:**
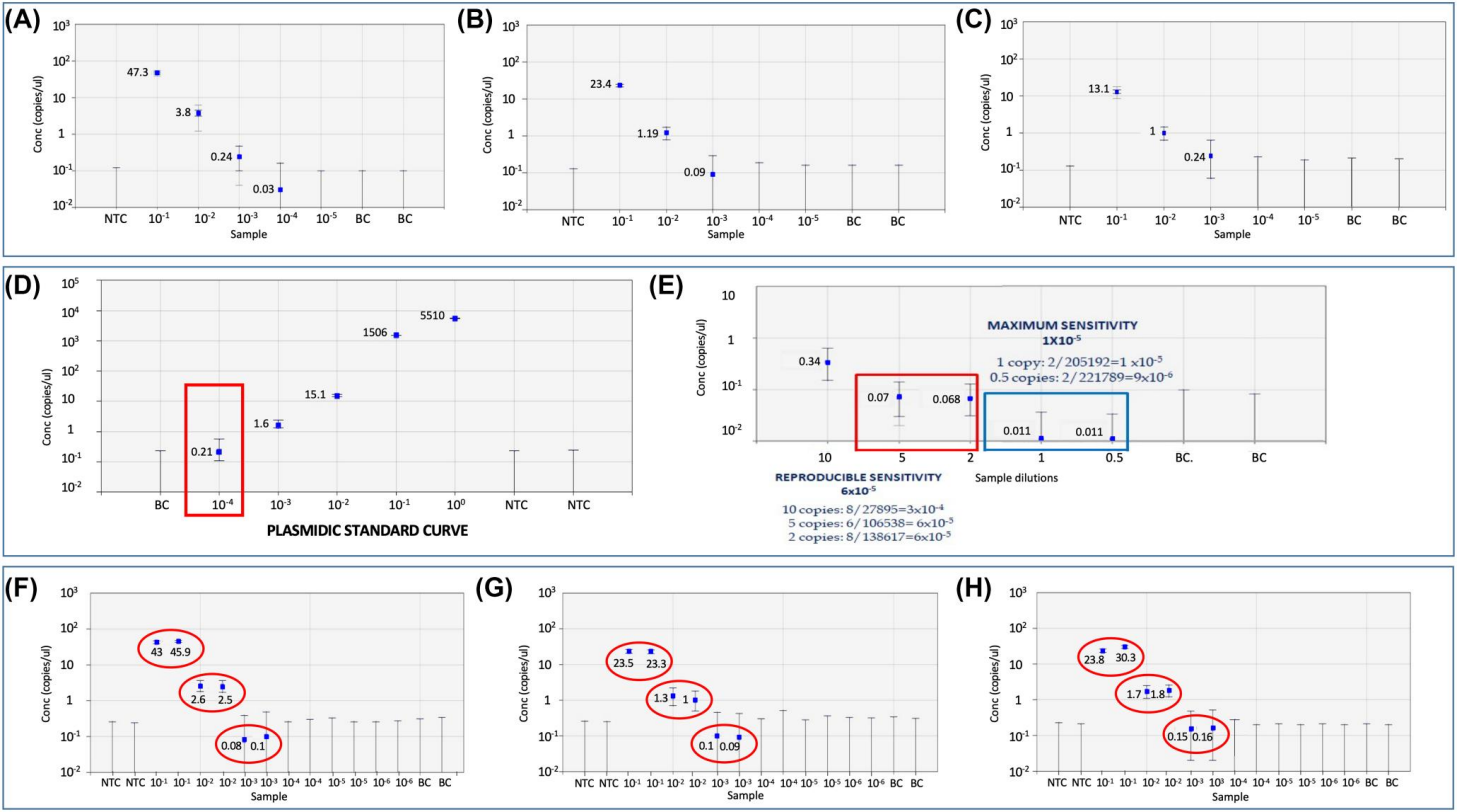
Evaluation of different cDNA amount: (A) 5 μL of cDNA input; (B) 2.5 μL of cDNA input and (C) 1 μL of cDNA input. Assessment of limit of detection (LOD): illustration of the maximum sensitivity and reproducible sensitivity achieved (D) compared to a standard curve (E). Assessment of reproducibility of the assay between replicates of dilution points and independent runs (F, G, H). Error bars are used to indicate the estimated error in a measurement. The length of an error bar indicates the uncertainty of the value. For an average value, a long error bar means that the concentration of the averaged values is low, so the average value is uncertain. In the graph, similar error bars are reported in samples where no positive events were detected and therefore no concentration was calculated

Preliminary experiments showed that using 5 μl of undiluted cDNA were adequate to quantify all serial dilutions, except for 10^−5^ (Figure 1A). The number of copies/μl progressively decreased and they were equal to 43.7, 3.8, 0.24 and 0.03 for the 10^−1^, 10^−2^, 10^−3^ and 10^−4^ respectively, in agreement with the dilution performed. At, variance, when the starting material was 2.5 μl or 1 μl we were able to quantify up to a dilution of 10^−3^ (Figure 1B and 1C). These experiments indicate that only 5 μL of cDNA guarantees signal detection, even in cases with low levels of *BCR*/*ABL1* transcript prompting us to use this volume in all subsequent experiments, except for the evaluation of the diagnostic samples, for which we used only 1 μl of cDNA, to avoid saturation of the assay.

### 4.2 Evaluation of LOD, specificity and reproducibility

The experiments performed showed that at 1 × 10^−4^, all replicates scored positive (2/2), at 5 × 10^−5^ we observed 6 out of 6 positive replicates, at 1 × 10^−5^ we documented six out of 8 positive replicates; at variance, at 5 × 10^−6^ only two replicates out of 12 were positive and, finally, at 1 × 10^−6^ only 2 out of 14 replicates scored positive. These data showed that ddPCR allowed to reach a sensitivity of 5 × 10^−6^. Nonetheless, we defined the 1 × 10^−5^ as the maximum sensitivity since up to this level 75% of replicates scored positive (6/8)—as opposed to what observed at lower concentrations (Figure 1D and 1E). Importantly, for both p190 and p210 transcripts, we obtained comparable results.

With regard to specificity, all NTC and healthy donor pools replicates tested always proved negative for *BCR*/*ABL1* transcripts.

Furthermore, we also documented a high reproducibility between the replicates of the diagnostic samples and dilutions, defining as reproducible all values which fell within the same logarithm. Notably, independent experiments analyzing the same samples provided highly similar results between all replicates and runs, thus confirming the robustness of the method (Figure 1F, 1G and 1H). This aspect has been further evaluated at lowest dilution points (Figure S1).

### 4.3 Comparison between Q‐RT‐PCR and ddPCR values of diagnostic samples

After having established the reaction parameters, specificity and sensitivity of the assay, we focused on the analysis of Ph+ ALL samples previously studied by Q‐RT‐PCR. First, we analyzed the diagnostic samples to assess the strength of the assay. In every plate, we included—as negative controls—MNC from a pool from healthy donors, NTCs and a Ph− ALL sample; we also included as positive controls three dilution points of the plasmid curve, which in addition to being a positive control are useful to assess the sensitivity achieved in each experiment. Each condition was run in triplicate. Overall, we analyzed 10 samples at diagnosis and we found a high degree of concordance (Table 1) between the results obtained with the two methods (Pearson correlation coefficient = 0.87). It is worth underlining that in Q‐RT‐PCR experiments cDNA was not diluted, while in ddPCR experiments, to avoid saturation issues, cDNA was diluted 1:5, thus explaining the apparent higher sensitivity of Q‐RT‐PCR.

**TABLE 1:** Comparison between Q‐RT‐PCR and ddPCR values of diagnostic samples

Total *n* = 10	Q‐RT‐PCR n copies (*BCR‐ABL1*/*ABL1*) × 100/μl	ddPCR n copies (*BCR‐ABL1*/*ABL1*) × 100/μl
DX1 (p210)	109.3	78
DX2 (p210)	126.9	109
DX3 (p190)	71.86	84
DX4 (p190/p210)	0.08/85.61	0.05/87.98
DX5 (p190)	81	71
DX6 (p210)	103	80
DX7 (p190)	75	88
DX8 (p210)	103.6	77
DX9 (p190)	67.5	79
DX10 (p190)	63.6	64

Abbreviations: ddPCR, digital droplet polymerase chain reaction; DX, diagnostic sample; Q‐RT‐PCR, quantitative real‐time polymerase chain reaction.

### 4.4 Comparison between Q‐RT‐PCR and ddPCR values of follow‐up samples

Next, we focused on the screening of samples with low levels of disease, classified as positive (*n* = 10), PNQ (*n* = 54) and negative (*n* = 24) by Q‐RT‐PCR analysis (Data S1). For eight patients, it was possible to evaluate longitudinally more follow‐up time points. Our experiments revealed a 100% concordance among the 10 positive cases by Q‐RT‐PCR, which proved positive also by ddPCR; at variance, within the 54 PNQ samples by Q‐RT‐PCR, 29 scored positive and quantifiable (54%), 13 were PNQ (24%) and 12 scored negative (22%) by ddPCR. Finally, within 24 negative cases by Q‐RT‐PCR, 13 were confirmed as such also by ddPCR (54%) while four were considered as PNQ (17%) and seven proved positive and quantifiable by ddPCR (29%). Results are summarized in Table 2. Thus, the overall concordance between the two assays was 41%. Importantly, ddPCR allowed to recover the rate of quantifiable samples in 46% of cases (*p* < 0.0001).

**TABLE 2:** Comparison between Q‐RT‐PCR values and ddPCR of follow‐up samples

Total *n* = 88 samples		Q‐RT‐PCR		
		POS (*n* = 10)	PNQ (*n* = 54)	NEG (*n* = 24)
ddPCR	POS	10 (100%)	29 (53.7%)	7 (29.1%)
	PNQ	‐	13 (24.1%)	4 (16.7%)
	NEG	‐	12 (22.2%)	13 (54.2%)

Abbreviations: ddPCR, digital droplet polymerase chain reaction; NEG, negative; PNQ, positive non‐quantifiable; POS, positive; Q‐RT‐PCR, quantitative real‐time polymerase chain reaction.

## 5 DISCUSSION

ddPCR is an innovative method that is being studied and standardized at an international level, within the EuroMRD Consortium in the Digital group, of which we are members. The applicability of ddPCR has been explored in several fields, including haematological malignancies, particularly in Ph− ALL, in multiple myeloma, in mantle cell lymphoma, by monitoring IG/TR gene rearrangement and in follicular lymphoma by monitoring BCL2/IGH rearrangement.^23^, ^24^, ^25^, ^26^ These studies have shown that ddPCR is a sensitive and reliable MRD monitoring method, at least comparable to RQ‐PCR, regardless of the availability and/or amount of diagnostic material. These studies also showed that this approach allowed to recover informative data in some cases. At variance, to date, only one report has compared Q‐RT‐PCR and ddPCR in Ph+ ALL.^33^ In our study, we aimed at developing a robust ddPCR assay for the correct quantification of both p190 and p210 *BCR/ABL1* transcripts, with the final goal of evaluating if this technique might refine Q‐RT‐PCR ‐based MRD evaluation. We first defined the analytical parameters to investigate the methodological applicability of this new technique to *BCR/ABL1* transcripts, establishing the required amount of cDNA at diagnosis and in MRD samples, the LOD, the specificity and the reproducibility. The results showed that our ddPCR *BCR/ABL1* assay had a sensitivity between 1 × 10^−5^ and 5 × 10^−6^, allowing to detect also low levels of transcripts, and was specific since no amplification was observed in any of the negative controls. The rate of reproducibility was very high between the replicates within the same run and between independent experiments.

After having set the experimental procedures, we used this approach in the screening of diagnostic and follow‐up samples from Ph+ ALL patients, the majority enrolled in the recently published GIMEMA LAL2116 trial,^12^ in which MRD was evaluated by Q‐RT‐PCR at various time points. We tested 10 diagnostic samples and 88 follow‐up samples. The analysis of the samples at the onset of the disease did not show differences between Q‐RT‐PCR and ddPCR approaches, and we observed a high degree of concordance between the results obtained with the two methods, probably as a consequence of the high disease burden. We then focused on the screening of different MRD time points and in particular on samples that were classified as PNQ or that proved negative by Q‐RT‐PCR analysis. We introduced in the screening also the negative cases since we aimed at investigating whether by increasing the sensitivity of the method we do not lose in specificity, with the risk of incurring in false positive results, that may lead to inappropriate therapeutic decisions. The study showed that ddPCR can reduce the proportion of PNQ samples, that represents a grey zone in the clinical practice, compared to RQ‐PCR, increasing significantly the proportion of quantifiable samples (46% of cases, *p* < 0.0001). Importantly, from a clinical standpoint, of the seven samples ‐ corresponding to five patients—that were negative by Q‐RT‐PCR and positive by ddPCR during follow‐up, 4/5 experienced a relapse (2 hematologic and 2 CNS).

In the study by Coccaro and colleagues,^33^ the comparison between Q‐RT‐PCR and ddPCR was performed only in 20 samples (including seven positive, 10 PNQ and three negative cases); besides confirming the positivity of the seven positive cases, the authors showed that ddPCR was able to quantify the disease in nine out of 10 PNQ samples, while one PNQ resulted negative. The three negative cases resulted positive by ddPCR, thus representing the major discrepancy between Q‐RT‐PCR and ddPCR results, indicating that data interpretation between this study and the one from Coccaro et al. is different: as a matter of fact, the authors decided to consider as quantifiable also a single positive droplet.

The samples evaluated in this trial derive from patients enrolled in the GIMEMA 2116 trial, based on a chemo‐free strategy, in which patients received dasatinib (plus steroids) in induction, followed by a consolidation phase with the bispecific monoclonal antibody blinatumomab: the preliminary results (median follow‐up: 18 months) of this trial are extremely promising, with OS and DFS of 95% and 88%, respectively.^12^ With this relatively short period of observation, so far no differences in survival have been observed between patients in CMR and PNQ. A longer follow‐up is necessary to document if this more sensitive MRD evaluation may have a clinical impact and thus translate into a further refinement in the prognostic stratification of adult Ph+ ALL patients. This is a key point as many patients with a good biologic profile at diagnosis and follow‐up may be managed without systemic chemotherapy and allogeneic transplant.

To our knowledge, our study provides for the first time a recommendations for the use of ddPCR analysis for adult *BCR*/*ABL1*+ ALL cases, showing that ddPCR allows to recover the quantifiability of MRD in a large proportion of patients, who otherwise would fall into a non‐quantifiable range of Q‐RT‐PCR, and launch the bases for using this approach also in Ph+ ALL. Last, but not least, ddPCR is being set up also for the evaluation of detrimental mutations, that is, T315I mutations, for which a rapid switch in treatment is pivotal for avoiding full‐blown relapses. In the forthcoming future, ddPCR might thus improve the overall clinical management of Ph+ ALL patients.

## CONFLICT OF INTEREST

No conflict of interest to report.

## AUTHORS CONTRIBUTION

Michela Ansuinelli, Irene Della Starza and Alessia Lauretti performed experiments, analyzed data and wrote the manuscript; Loredana Elia, Veronica Siravo, Monica Messina, Lucia Anna De Novi and Martina Canichella performed experiments; Anna Guarini and Robin Foà designed the research and critically revised the manuscript, Sabina Chiaretti designed the research, provided clinical data, analyzed data and critically revised the manuscript.

### PEER REVIEW

The peer review history for this article is available at https://publons.com/publon/10.1002/hon.2913.

## Supporting information

Supporting Information S1

Supporting Information S2

## Data Availability

The data that support the findings of this study are available from the corresponding author upon reasonable request.
