# Supplementary material for: Applicability of droplet digital polymerase chain reaction for minimal residual disease monitoring in Philadelphia‐positive acute lymphoblastic leukaemia
Source: Hematol Oncol. 2021 Aug 16;39(5):680–6. doi: 10.1002/hon.2913 (PMC9292453; doi:10.1002/hon.2913)
Supplement: Supplementary file 1 — Supporting Information S1 [file HON-39-680-s002.docx]

**SUPPLEMENTAL MATERIALS**

**Population of study**

Within patients enrolled in the GIMEMA LAL2116 trial, positive cases included 1 sample at day +22, 1 sample at day +85, 1 sample post cycle 1 of blinatumomab, 1 sample post cycle 2 of blinatumomab, 1 sample post cycle 3 of blinatumomab, 1 sample post cycle 4 of blinatumomab and 4 follow-up samples. PNQ samples included 2 samples at day+22, 3 at day +45, 4 at day +57, 5 at day +85, 6 post cycle 1 of blinatumomab, 3 post cycle 2 of blinatumomab, 4 post cycle 3 of blinatumomab, 4 post cycle 4 of blinatumomab, 3 post cycle 5 of blinatumomab and 14 follow-up samples and 4 post-allogeneic hematopoietic stem cell transplant (allo-SCT). Negative cases included 2 post cycle 1 of blinatumomab, 1 post cycle 2, 3 post cycle 3 of blinatumomab, 2 post cycle 5 of blinatumomab, 10 follow-up samples and 1 post-allo-SCT.

Within samples not belonging to the above-mentioned trial, there was 1 PNQ sample and 5 negative samples.

**DdPCR results interpretation**

For ddPCR results interpretation we followed the guidelines proposed within the EuroMRD Consortium (26):

MRD positive sample: a merge of events ≥3, regardless of the number of positive replicates.

MRD negative sample: all acceptable replicates with a merge of no or only one event.

MRD below quantifiable levels (BQL/PNQ) sample: a merge of event =2.

**Supplementary Figure.** Assessment of reproducibility of the assay between replicates of low levels (up to 10^-5^) dilution points in three different diagnostic samples analyzed in independent runs.
