# Supplementary figures and images for: Applicability of droplet digital polymerase chain reaction for minimal residual disease monitoring in Philadelphia‐positive acute lymphoblastic leukaemia
Source: Hematol Oncol. 2021 Aug 16;39(5):680–6. doi: 10.1002/hon.2913 (PMC9292453; doi:10.1002/hon.2913)

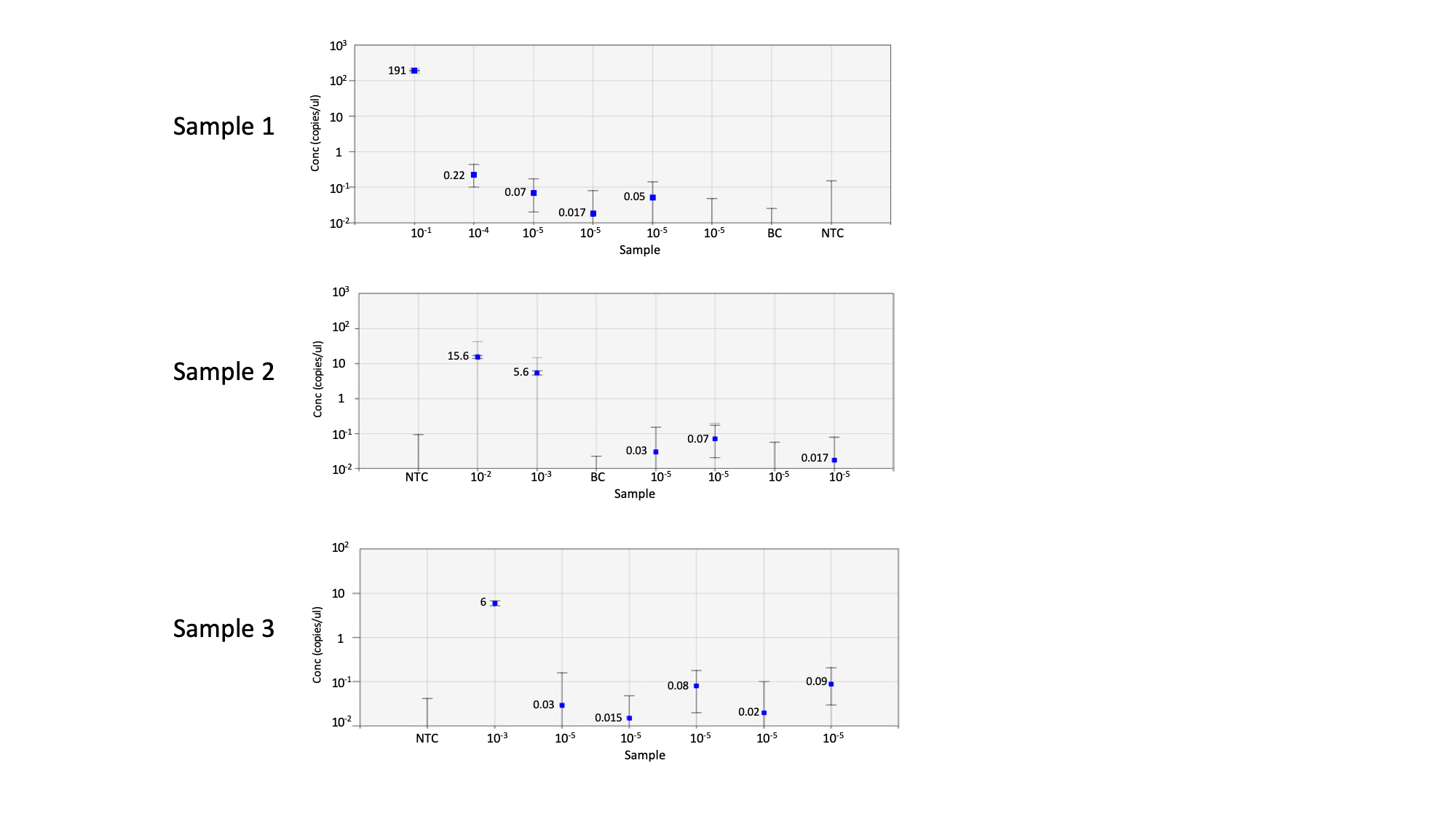

Supplement: Supplementary file 2 — Supporting Information S2 [file HON-39-680-s001.tiff]
